# Large-scale *k*-mer-based analysis of the informational properties of genomes, comparative genomics and taxonomy

**DOI:** 10.1371/journal.pone.0258693

**Published:** 2021-10-14

**Authors:** Yuval Bussi, Ruti Kapon, Ziv Reich

**Affiliations:** 1 Department of Biomolecular Sciences, Weizmann Institute of Science, Rehovot, Israel; 2 Department of Computer Science and Applied Mathematics, Weizmann Institute of Science, Rehovot, Israel; 3 Department of Molecular Cell Biology, Weizmann Institute of Science, Rehovot, Israel; University of North Carolina at Chapel Hill, UNITED STATES

## Abstract

Information theoretic approaches are ubiquitous and effective in a wide variety of bioinformatics applications. In comparative genomics, alignment-free methods, based on short DNA words, or *k*-mers, are particularly powerful. We evaluated the utility of varying *k*-mer lengths for genome comparisons by analyzing their sequence space coverage of 5805 genomes in the KEGG GENOME database. In subsequent analyses on four k-mer lengths spanning the relevant range (11, 21, 31, 41), hierarchical clustering of 1634 genus-level representative genomes using pairwise 21- and 31-mer Jaccard similarities best recapitulated a phylogenetic/taxonomic tree of life with clear boundaries for superkingdom domains and high subtree similarity for named taxons at lower levels (family through phylum). By analyzing ~14.2M prokaryotic genome comparisons by their lowest-common-ancestor taxon levels, we detected many potential misclassification errors in a curated database, further demonstrating the need for wide-scale adoption of quantitative taxonomic classifications based on whole-genome similarity.

## Introduction

Information theory, initially developed for the mathematical analysis of communication systems by Shannon [1], has been applied to molecular biology for decades. Gatlin’s pioneering works in the late 1960s were the first to define life as an information processing system [2, 3]. Since then, information-theoretical approaches have been used in a wide variety of biological sequence analyses, such as in the study and prediction of protein structure, protein-protein interactions, transcription factor binding motifs, gene identification, as well as for sequence assembly, alignment, and comparisons (see reviews [4–7]). As such, the application of information theory to biological sequences, concomitant with developments in sequencing technology and computational processing, has been foundational to the burgeoning field of bioinformatics. Within this field, a significant area of investigation is naturally devoted to the genome, wherein all of the hereditary information necessary to build and maintain an organism is stored. Specifically, the aforementioned mathematical framework lends itself towards the main foci of this work: quantitative characterization of global genomic information, whole-genome comparisons, and taxonomic classification.

Biological sequences are commonly analyzed using informational entropy-based quantitative methods, *e*.*g*. Shannon entropy, mutual information, Kullback-Leibler divergence, Renyi entropy, diffusion entropy, topological entropy, *etc*., and their estimators [8–17]. The Shannon entropy [1] of nucleotide distributions has long been studied as a predictor of genomic sequence functionality and taxonomy [18–22]. Similarly, the distributions of DNA words (*i*.*e*. oligomers of length *k*–also known as *k*-mers, n-tuples, n-grams) within long fragments of DNA has been shown to be highly characteristic of an organism [23, 24]. Thus, by using extracted short *k*-mers, sufficiently long DNA sequences could be taxonomically classified to different genomes efficiently, a common task in processing metagenomic data. Currently, tetranucleotide frequencies are used in the most popular tools for this process of binning metagenomic sequences [25, 26], however, longer lengths of *k* have been shown to improve the resolution of taxonomic classification. Alsop and Raymond compared mononucleotide through nonanucleotide (k = 1–9) signatures of 1424 microbes’ completed genomes and concluded that using heptanucleotides (k = 7) was the optimal compromise between accuracy and computation time [27].

While large-scale entropy-based genomic analyses using *k*-mer frequencies with *k* > 9 have been computationally impractical thus far, alternate methods which are related to or estimate entropy based on sets of distinct *k*-mers have been successful for larger values of *k*. Sequence space coverage (SSC) is one such metric which has been applied to assess genome complexity [14]. It is obtained by counting the *k*-mers in a sequence and then dividing the number of distinct *k*-mers observed by the total number of possible *k*-mers. SSC can be calculated both exhaustively or through efficient (sub-linear) sampling-based methods. Using these methods, Liu et al. [14] analyzed the informational properties of seven organisms, including both eukaryotes and prokaryotes, and showed that > 98% of 12-mers and < 2% of 19-mers appeared in vertebrate genomes, meanwhile in *Escherichia coli*, the same range of sequence space coverages (> 98%, < 2%) were obtained at *k*-mer lengths 9 and 15, respectively. They concluded that sequence space coverage depends heavily on genome length and GC content. However, they did not explore normalization for these parameters. They also showed that, for *k*-mer lengths between 15 and 20, the human genome is significantly more compact in sequence space than a random genome and proposed that a large set of 15-mers could be used as probes to detect non-human DNA in samples [14].

Currently, the use of higher-length *k*-mers (k>10) is ubiquitous in bioinformatics algorithms for genome assembly and comparisons [28, 29]. Methods using word statistics are often referred to as “alignment-free” (AF) methods, in contrast to traditional alignment algorithms, such as BLAST [30]. While alignment methods can be computationally demanding for large-scale comparisons [31], AF approaches have been extremely successful at efficiently computing them [32]. One such approach is to compute pairwise matrices of similarity (or distance), from sets of distinct *k*-mers, by the Jaccard similarity index [33], a metric defined as the cardinality, or number of distinct elements, of the intersection over the cardinality of the union of two sets (A, B):

$$JS(A,B)=\frac{\left|A\cap B\right|}{\left|A\cup B\right|}$$ (1)

The Jaccard similarity approximates the average nucleotide identity (ANI) metric [34], which can be computed by several alignment-based algorithms, which are themselves computational approximations of the DNA-DNA hybridization (DDH) molecular biology technique [35, 36] classically used for comparing genomes and for taxonomic classification. A 70% DDH value, which correlates to a ~95% ANI value, has been considered a gold standard threshold for defining prokaryotic species, and this threshold is especially valuable for correctly classifying prokaryotes with a high sequence similarity (>97%) between their 16S rRNA marker genes [37–42]. Under the Poisson distribution model, the ANI between two sequences, A and B, relates to the Jaccard similarity of their constituent *k*-mer sets by the following equation:

$$\frac{ANI(A,B)}{100}=1+\frac{1}{k}*\ln\left(\frac{2*JS(A,B)}{1+JS(A,B)}\right)$$ (2)

where *k* is the *k*-mer length [43, 44].

The most prominently used AF genome comparison algorithms, Mash [44] and FastANI [43], employ the efficient MinHash technique [45] for quick estimation of the similarity of two sets. This technique reduces large sequences (or sequence sets) to compressed sketch representations, containing a fixed-sized subset of *k*-mers, and estimates the Jaccard similarity index, and thereby ANI, from these smaller sketches. Both tools require defining the length of *k* as well as the size of the sketch. Mash, with a default *k* = 21, and FastANI (used for microbial genomes only), with a default *k* = 16, have successfully clustered tens of thousands of genomes in less than 100 CPU hours [43, 44]. These large-scale, pairwise, whole-genome comparisons have demonstrated an intraspecies-level boundary at approximately 95% similarity, as was previously reported by the alignment-based ANI.

Higher levels of taxonomy above species (genus, family, *etc*.) are not well demarcated by the methods discussed above, possibly because these methods are less accurate with increasing divergence. The *k*-mer length used affects the resolution, precision, and bias of similarity estimates; relatively low lengths have been shown to result in many shared, non-homologous *k*-mers (aka *k-mer homoplasy*), which has largely been considered as noise in phylogenetic reconstruction [46]. Utilizing multiple *k*-mer sizes to fit *k*-mer “palettes” has been shown to improve strain-level accuracy of taxonomic profiling [47]. Nonetheless, hierarchical clustering of pairwise similarity matrices using a single *k*-mer size has been successfully used to approximate phylogenetic relatedness and recapitulate phylogenetic trees. Fan *et al*. reconstructed the phylogeny of 12 mammals and 21 tropical tree genomes using *k*-mer sets from raw sequencing reads (*k* = 21 and 27 respectively) [46]. Ondov *et al*. demonstrated Mash’s ability to approximate the phylogenetic tree for 17 primates [44]. Bernard and Ragan used another alignment-free measure, a variant of the D2 statistic, with 25-mers to generate a network of phylogenetic relatedness for 143 bacterial and archaeal genomes [48].

Large uncurated taxonomic databases are known to have many incorrect labels, which may affect many downstream bioinformatic applications and evolutionary studies. Very recently, Parks *et al*. have used similar AF approaches, such as Mash and FastANI, on tens of thousands of microbial genomes to propose a standardized bacterial taxonomy database (Genome Taxonomy Database) defining representative species clusters and improving the classification of uncultured bacteria [49, 50].

While alignment-free methods for genome comparisons continue to be an active area of bioinformatics research [29, 51], not much attention has been given to the optimal length of *k* and how it relates to global genomic properties and taxonomic signals at different levels of relatedness. Many of the works reviewed either defined the length of *k* empirically, according to computational limits, or, in most cases, did not discuss the choice at all. In this work, we sought to characterize global informational properties of all complete genomes, listed in one of the only curated databases, KEGG [52], by assessing their sets of constituent *k*-mers with alignment-free methods, capitalizing on the availability of high-throughput next-generation sequencing data and high-performance computing (HPC) resources. In doing so, we aimed to identify optimal word lengths that can be used for whole-genome comparisons, taxonomic profiling and other genomic applications.

## Methods

### Genome and taxonomy data retrieval

We downloaded all 5805 complete genomes listed in the KEGG GENOME database (https://www.genome.jp/kegg/catalog/org_list.html) [52] as of March 10, 2019. FASTA files for all genomes were retrieved from the National Center for Biotechnology Information (NCBI) genome databases, RefSeq [53] and GenBank [54]. Taxonomic hierarchy information was retrieved from the NCBI taxonomy database [55] via the *myTAI* R package [56]; for each organism we retrieved all available labels for its taxonomic levels (*i*.*e*. species, genus, family, order, class, phylum, superkingdom, *etc*). The data and code are available at https://github.com/zreichlab/LargeScaleKmerAnalysis.

### *k*-mer counting and genome statistics

Each genome was processed with the software *KMC3*, a disk-based program for counting *k*-mers [57]. All *k*-mers were extracted from the sequences, processed from left to right with a sliding window of chosen length, and output to a compact “KMC” database as described in Deorowicz *et al* [58]. In addition, statistics such as the sequence length and total number of distinct *k*-mers, *i*.*e*. *k*-mers that occur at least once, were also output by the software. [Note, we use the term ‘unique’ to refer to *k*-mers that occur only once, aka singletons or depth-1 *k*-mers, however the software refers to distinct *k*-mers as unique. Since the direction of genomic sequences is often unknown, a *k*-mer and its reverse complement is, by default, considered identical, and thus only the *canonical k*-mer, the lexicographically smaller from each pair, is recorded.] The KMC database for each genome contains the set of all of the distinct observed canonical *k*-mers, as well as their frequencies, *i*.*e*. the sum of the frequency for the canonical *k*-mer and its reverse complement. The *k*-mer counting process was performed for lengths of *k* ranging from 3 to 51. As is common practice in many bioinformatic applications, only odd lengths of *k* were considered in order to reduce the computational burden and simplify accounting for complementarity by avoiding palindromes [59], although their effect on *k*-mer counts is likely negligible at large scale. GC content of the genomes was computed using the Seqtk toolkit (https://github.com/lh3/seqtk) [60]. Correlation and regression analyses were performed in MATLAB [61].

### Random sequence analysis

We analyzed 110 pseudorandom sequences, henceforth referred to as random sequences, generated with the MATLAB randseq function (MATLAB and Statistics Toolbox Release 2019b) [61]. Generated sequences varied in length from 100kbp to 10Gbp for a total of ~167Gbp (10 sequences generated at each length: 100kbp, 500kbp, 1Mbp, 5Mbp, …, 5Gbp, 10Gbp), thereby spanning the range of observed genome lengths in our dataset. We performed *k*-mer counting on the random sequences, as described above with *k* ranging from 3 to 51 odd, and found that the number of distinct *k*-mers was comparable to that expected for a simple random sampling with replacement, << 0.01% difference from the mean for all lengths measured, given by the following equation:

$$E[N_{D,R}]=S\left(1-{\left(1-\frac{1}{S}\right)}^{N_{T}}\right)=\frac{4^{k}}{2}\left(1-{\left(1-\frac{2}{4^{k}}\right)}^{N_{T}}\right)$$ (3)

where *E*[*N*_*D*,*R*_] is the expected number of distinct *k*-mers for a random sequence, *N*_*T*_ is the total number of *k*-mers, and $S=\frac{4^{k}}{2}$ is the total number of possible *k*-mers for length *k*, *i*.*e*. the size of the canonical *k*-mer sequence space. The total number of *k*-mers, *N*_*T*_ = *L*−*k*+1(*L*≫*k*), is given by the length of the sequence, *L*, minus a small term to account for symbols at the end of the sequence where the sliding window is not fully covered. This assumes that a genome is a single unified sequence, which is not always the case; for genomes with multiple chromosomes or disjoint sequences, *N*_*T*_ = *L*−n*k+*n*, where *L* is now the sum of all sequence lengths and *n* is the number of sequences, (*L*≪n*k).

### Sequence space coverage

Sequence space coverage (SSC) is defined as the number of observed distinct canonical *k*-mers, *N*_*D*_, divided by the number of possible canonical *k*-mers, $S=\frac{4^{k}}{2}$, for given length *k*:

$$SSC=\frac{{2N}_{D}}{4^{k}}$$ (4)

It was desirable to normalize the SSC by a factor relative to the length of the sequence, as we observed that *N*_*D*_, and thus SSC, largely depend on the total number of *k*-mers, *N*_*T*_, as well as because the SSC decreases exponentially with increasing lengths *k*. We thus divided the observed SSC (Eq 4) by the expected SSC for a random sequence of the same length to obtain normalized SSC (NSSC), equivalent to the observed number of distinct *k*-mers, *N*_*D*,*O*_, divided by the expected number of distinct *k*-mers for a random sequence, *E*[*N*_*D*,*R*_] (Eq 3):

$$NSSC=\frac{SSC_{O}}{E[{SSC}_{R}]}=\frac{N_{D,O}}{E[N_{D,R}]}=\frac{N_{D,O}}{S(1-{\left(1-\frac{1}{S}\right)}^{N_{T}})}=\frac{2N_{D,O}}{4^{k}(1-{\left(1-\frac{2}{4^{k}}\right)}^{N_{T}})}$$ (5)

In order to know at which length of *k* the observed sequence differs in SSC most from that expected of an equally long random sequence, we computed the *k* where the minimum NSSC occurs across all possible values of *k* (3 to 51 odd):

$$\underset{k\in \{3,5,\ldots ,51\}}{\mathrm{argmin}}NSSC(k)=\underset{k\in \{3,5,\ldots ,51\}}{\mathrm{argmin}}\frac{{2N}_{D,O}}{4^{k}(1-{\left(1-\frac{2}{4^{k}}\right)}^{N_{T}})}$$ (6)

### Genome comparisons and analysis

KMC databases extracted from genomes can be compared using set operations (union and intersection) via the *KMC tools* functions [57]. The cardinalities of the union and intersection of sets are related as follows:

|A∩B|+|A∪B|=|A|+|B| (7)

Thus, once the union of two *k*-mer sets is computed, obtaining the cardinality of the intersection, is simple:

|A∩B|=|A|+|B|−|A∪B| (8)

For computing the pairwise matrix of similarity (or distance), using the Jaccard similarity index (Eq 1), we only needed to compute the union, or equivalently the intersection, of *k*-mer sets for all pairs of genomes.

In order to observe the relationships between clusters of genomes, we computed the hierarchical agglomerative clustering of the pairwise similarity matrix followed by optimal leaf ordering. For the clustering, we used the hclust function in R (stats v3.6.2) with the ward.D2 method [62]. The resulting dendrogram was reordered to minimize the distance between neighboring leaves by the optimal leaf ordering method of Bar-Joseph *et al*. [63], as implemented in the reorder_hclust function (seriation v1.2-8) [64]. Trees were plotted with the plot.phylo function from the ape package [65]. Side-by-side comparison of the tree to reference was computed and visualized with the tanglegram function (dendextend v1.13.4) [66]. A generalized Robinson-Foulds tree distance was computed with the treedist package (v2.1.1) [67]. Random trees were generated with the rtree function (ape v5.4) [65]. Reference trees were generated in Newick format based on the major taxon levels (superkingdom, phylum, class, order, family, genus, species) [68]. For the Mammalian subtree, intermediate taxon labels (*e*.*g*. magnorder, superorder, grandorder, *etc*.) were included as well to resolve polytomies. The remaining ten nodes that still contained three branches were resolved by searching these specific taxon groups for phylogenetic trees published in the literature [69–78]. In one case, relevant for the treeshrew genus, *Tupaia*, there were conflicting trees hypothesized and published in the literature for the placement of its order (Scandentia) in relation to primates and rodents [79, 80]. We defaulted to the traditionally accepted Euarchonta grandorder which places Scandentia sister to Primatomorpha, although this placement has been disputed [78]. Branch lengths were computed with the compute.brlen function (ape v5.4) [65] and are thus not meaningful for the reference trees.

### Analyzing similarity by taxon level

Similarity scores were labelled and grouped by the taxon level (organism, superkingdom, phylum, class, order, family, genus, species) of the lowest-common-ancestor (LCA) for each prokaryotic pairwise comparison. Distributions of the log-transformed Jaccard similarity for different taxon levels were computed by kernel density estimation using the ksdensity function in MATLAB [61] with bounded support from -8 to 0, *i*.*e*. bounded from 10^-8^ to 1 in Jaccard similarity. 1177 pairs, out of the total ~1.3M, that shared zero *k*-mers were excluded for estimation of the distribution. For each genome, the median and maximum similarity score was also computed at each LCA taxon level where at least one relative existed in the database. In the ideal case, every genome would have a higher similarity (lower distance) score to those from lower (closer) LCA taxon levels on average, *i*.*e*. a genome would have higher similarity to the group of genomes whose LCA is at the genus-level compared to those whose LCA is at the family-level, *etc*., as described by:

$$median\{s(x,y)|y\in LCA(x,r_{1})\}>median\{s(x,y)|y\in LCA(x,r_{2})\},\;r_{1}<r_{2}$$ (9)

where *s(x*,*y)* is a function that returns a similarity score between genomes *x* and *y* and *LCA(x*,*r)* is the set of all relatives with an LCA *r* taxon levels away from *x*; a lower *r* is a closer taxon level, and for strains of the same species *r* = 1. For each genome, we computed the difference of median log-transformed Jaccard similarity for consecutive taxon level pairs such that a delta less than zero would indicate violation of the equation above. Plots and visualizations were generated with MATLAB.

### High performance computing

The *k*-mer counting and genome comparison computations, outlined in their respective sections, were run on the high-performance computing (HPC) cluster of the Weizmann Institute.

## Results

5805 genomes and their taxonomic hierarchy information were retrieved from the curated KEGG GENOME database and NCBI as described in the Methods. The 5805 organisms represented 3986 species, 1634 genera, 701 families, 382 orders, 190 classes, 88 phyla, and 3 superkingdom domains (474 Eukaryota, 5044 Bacteria, and 287 Archaea). Of the eukaryotes, there were 201 animals, 98 plants, 125 fungi, and 50 protists.

### Exploring the informational properties of genomes

**Fig 1:**
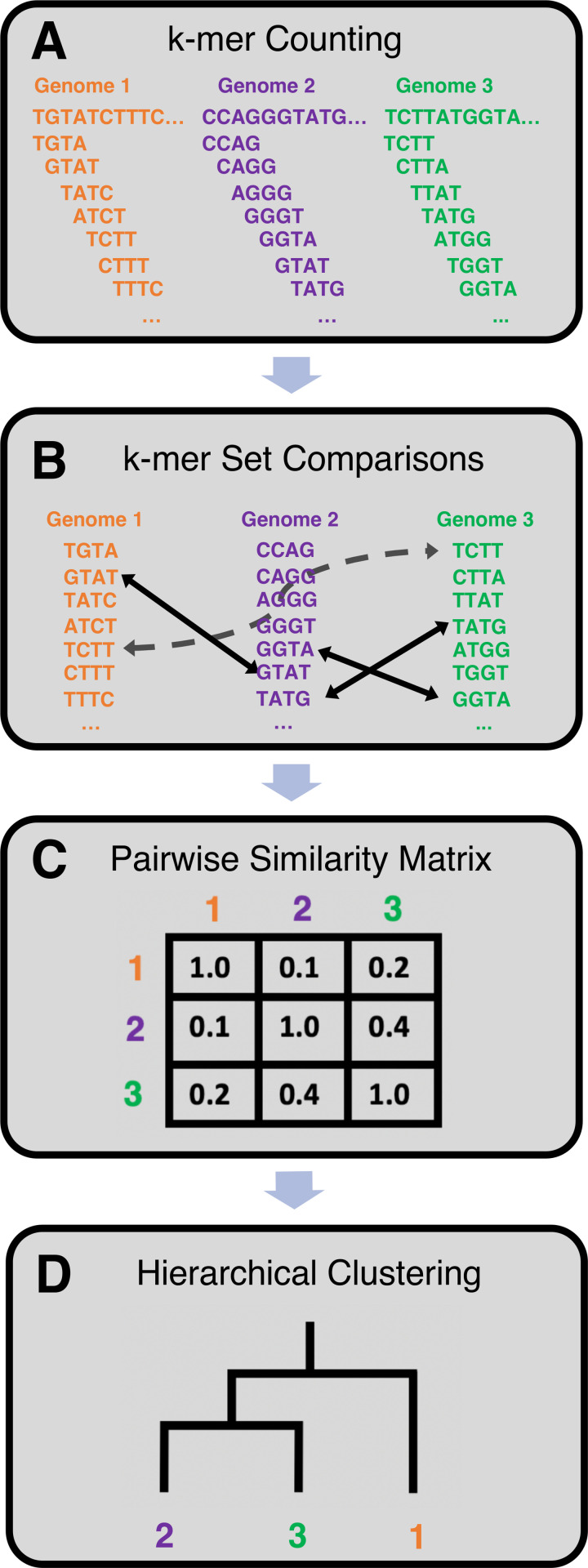
Workflow for alignment-free genome comparisons used in this work. (A) Genomes are processed from left to right with a sliding window of fixed length resulting in *k*-mer databases. As an example, *k*-mer extraction is shown for a short section of three DNA sequences with *k* = 4. (B) *k*-mer set comparisons, *e*.*g*. union and intersection, are then computed for pairs of genomes. Arrows indicate the *k*-mers shared between *k*-mer sets. (C) From the set comparisons, similarity scores are calculated resulting in a pairwise similarity matrix. (D) Hierarchical clustering of the similarity matrix yields a tree which can be compared to a reference phylogenetic tree.

**Fig 2:**
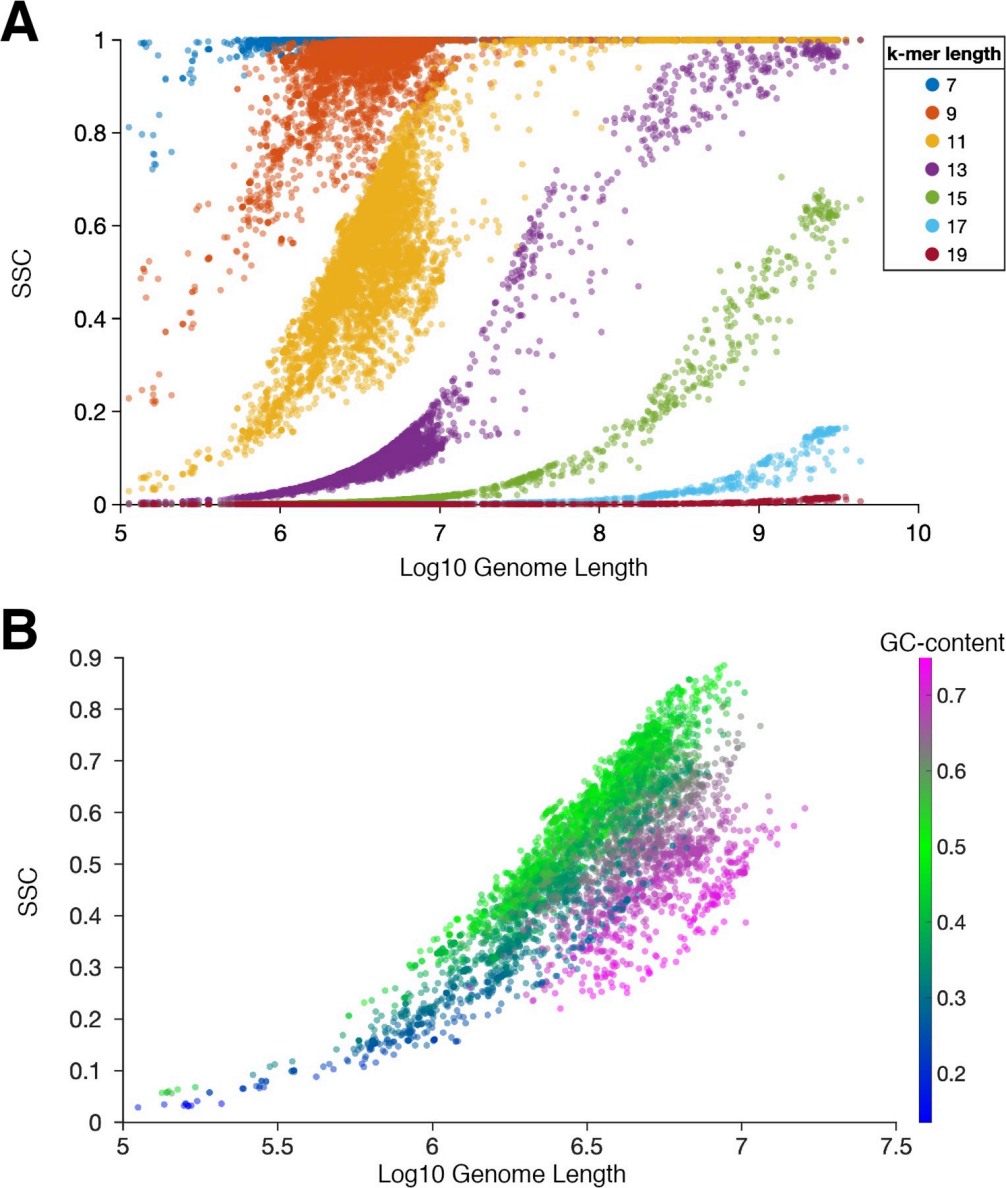
Sequence space coverage (SSC) largely depends on genome length and GC-content. (A) SSC for each genome is plotted for *k* ranging from 7 to 19 (odd). SSC exhibits a sigmoidal relationship with log-transformed genome length. (B) SSC of prokaryotic genomes, for *k* = 11 (gold color in panel A) is plotted with points colored by GC-content. GC-content has a moderately strong positive correlation with log-transformed genome length (change from blue to magenta color; Pearson correlation coefficient r = 0.606, p < 0.001). SSC decreases as GC-content differs from the random, 0.5 (bright green).

Genomes were analyzed with an alignment-free (AF) comparison workflow (Fig 1), similar to that recently described in Bernard *et al* [81]. *k*-mer counting (Fig 1A) was first performed to extract a compact database of distinct canonical *k*-mers, henceforward referred to as just *k*-mers, for each genome with *k* ranging from 3 to 51 (see Methods). Sequence space coverage (SSC) was then analyzed in order to determine at which length of *k* to perform subsequent *k*-mer set comparisons (Fig 1B). SSC for each genome, at each length *k*, was computed (Eq 4) and plotted for *k* ranging from 7 to 19 (Fig 2A). For lengths of *k* outside of this range, plotting SSC for single genomes was not very informative as either the sequence space was very small and completely covered (*k*<7) or very large with low coverage, less than 1%, undetectable in the plot (*k*>19). SSC exhibited a sigmoidal relationship with respect to log-transformed genome length, and, as expected, genomes with higher lengths tend to have higher sequence space coverage at each *k*. As the sequence space grows exponentially, with respect to *k*, and the total number of *k*-mers, for each *k*, is limited to the genome length, SSC diminishes rapidly with increasing *k* (Eq 4). The longest eukaryotic genomes in the database, with lengths greater than 10^9^ base pairs (1 Gbp), covered approximately 100%, 90%, 50%, 10%, and 1% of the 11-, 13-, 15-, 17-, and 19-mer sequence spaces, respectively. In comparison, the average bacterial genome with length ~4 Mbp covered approximately 100%, 90%, 50%, 10%, and 1% of the 7-, 9-, 11-, 13-, and 15-mer sequence spaces, respectively.

In order to examine the effect of GC-content on SSC, we plotted 11-mer SSC (Fig 2B) with points colored by GC-content. The 11-mer SSC was chosen for examination as it contained the most data points near the middle of the sigmoidal curve, below saturation and above very low SSC, and therefore provides a wide range for visualization. The plot was limited to 5,331 prokaryotic (bacterial and archaeal) genomes, since the longer eukaryotic genomes saturated the SSC at this length of *k* and, in general, have a narrower distribution of GC-content (eukaryotes: 0.42 ± 0.08 [mean ± SD], n = 474; prokaryotes: 0.49 ± 0.13, n = 5331). As seen in Fig 2B, for a given genome length, SSC decreases as the GC-content deviates away from 50% (bright green color). Multiple regression analysis to predict 11-mer SSC (Y) for these genomes based on log-transformed genome length (X_1_) and the absolute difference of GC-content from 0.5 (X_2_) generated a relation [Y = -1.8329 + 0.3839·X1 – 1.3073·X_2_; F(2, 5328) = 2.01 x 10^4^, p < 0.001] with an R^2^ of 0.883. GC-content has a moderate positive correlation with X_1_ (Pearson correlation coefficient r = 0.606, p < 0.001), consistent with the recent report of Almpanis *et al* [82]. However, after the absolute value transformation of GC-content to X_2_, there is only a very weak negative correlation with X_1_ (Pearson correlation coefficient r = -0.162, p < 0.001). Since prokaryotic GC-content ranges from 13.5% for *Candidatus Zinderia insecticola* to 74.9% for *Anaeromyxobacter dehalogenans 2CP-C*, X_2_ falls within the range of 0 to 0.365. In extreme cases, *i*.*e*. X_2_ = ~0.35 (corresponding to 15%, or equivalently 85%, GC-content), a genome is predicted to have a reduction of ~45% 11-mer SSC compared to a genome of equal length with 50% GC-content, or X_2_ = 0. As approximately 90% of prokaryotic genomes have GC-content between 30–70%, the X_2_ factor has smaller influence on SSC than X_1_ in practice, especially for higher lengths of *k* where the sequence space is much larger. In comparison, the aforementioned equation predicts a ~76% increase in 11-mer SSC for a genome length ~100X larger with the same GC-content (note that prokaryotic genome lengths range from 10^5^–10^7^ bp). As eukaryotic genomes in our dataset have even higher genome lengths, up to ~10^10^ bp, and less extreme GC-content, in subsequent SSC analyses we focus solely on the factor of genome length, and the sum of genome lengths.

**Fig 3:**
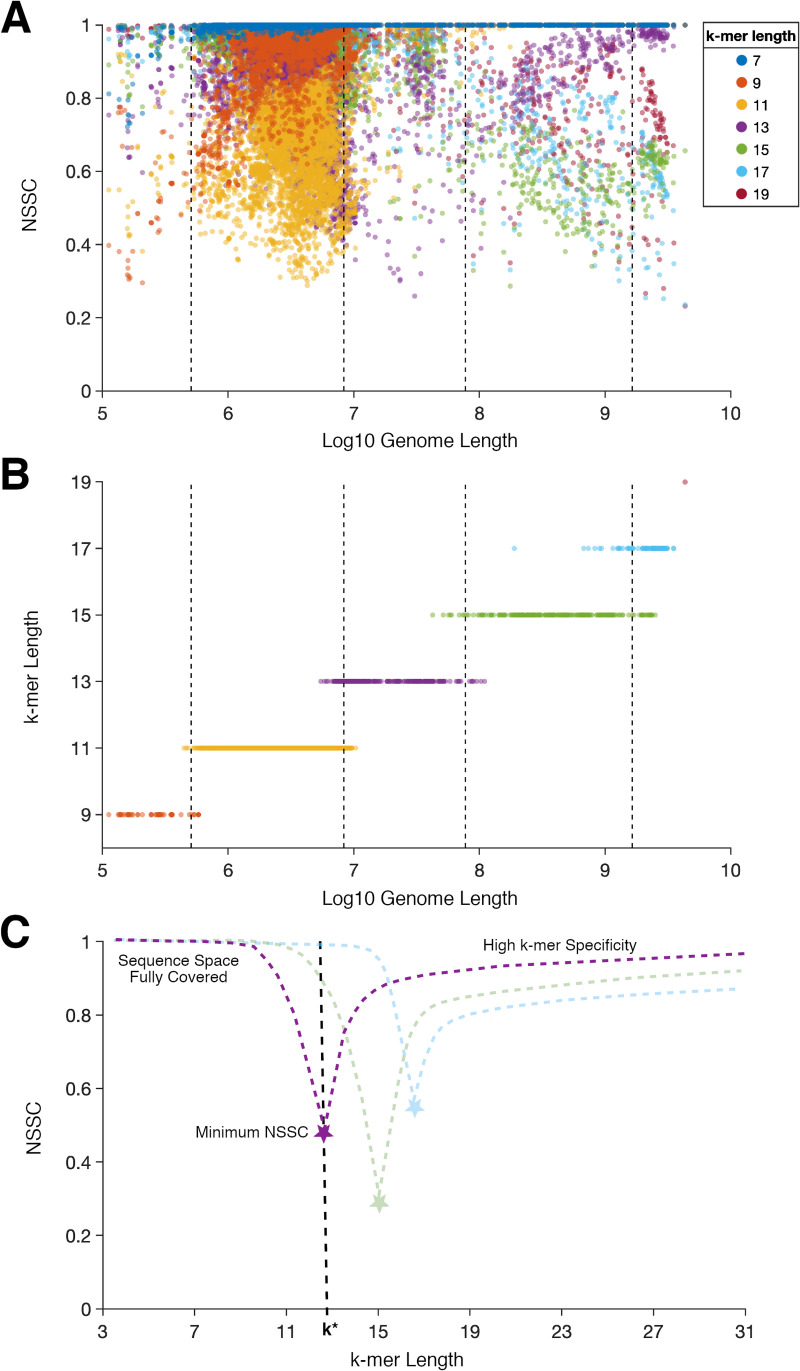
Normalized sequence space coverage (NSSC) of genomes attains a minimum within the range of *k* = 9–19. (A) SSC from Fig 2A was normalized by the expected SSC estimated for random sequences of the same length (Eq 5). (B) For each genome, the *k*-mer length at which it attains a minimum NSSC (Eq 6) was plotted against log-transformed genome length. Dotted lines in (A) and (B) represent thresholds (set at 0.5) from logistic regression to predict the *k*-mer lengths at which minimum NSSC will occur based on log genome length. (C) A schematic of NSSC curve for three idealized genomes of different lengths (purple, green and blue dotted lines; see S1A Fig for examples). At low *k*-mer lengths, NSSC is high due to full sequence space coverage. On the other hand, at high *k*-mer lengths, NSSC is also high due to high *k*-mer specificity. In the range of *k* = 9–19, NSSC attains a minimum at k*, denoted by star symbols, representing minimal entropy and maximum intragenomic shared *k*-mers relative to estimates for random sequences of the same length.

To further examine how SSC behaves for varying *k*-mer and genome lengths, we normalized SSC by the expected SSC estimated for random sequences of the same length. Analyzing 110 generated random sequences ranging in length from 100kbp to 10Gbp, we found that the expected number of distinct *k*-mers was comparable to simple random sampling with replacement (Eq 3; see Methods for further details). Next, we used this to normalize sequence space coverage for each genome, at each length *k* (Eq 5) and plotted the normalized sequence space coverage (NSSC) for *k* ranging from 7 to 19 (Fig 3A). For each genome, the *k*-mer length at which its minimum NSSC was attained (k*) was also recorded (Eq 6) and plotted (Fig 3B). k* ranged from 9 to 19 and generally increased with increasing genome lengths. For low values of *k*, the sequence space is completely covered, *i*.*e*. all possible *k*-mers are present in the sequence. In this case, both the observed SSC and that expected for a random sequence are 1.0, and accordingly the NSSC is also equal to 1.0. On the other hand, for high values of *k*, the vast majority of *k*-mers are unique, *i*.*e*. high *k*-mer specificity, and therefore both the observed SSC and that expected for a random sequence are approximately equal to the total number of *k*-mers divided by the size of the sequence space; this case also results in a NSSC equal to ~1.0. Within the range between these two extremes, NSSC attains a minimum (at *k* = k*), representing a maximum of intragenomic shared *k*-mers (minimum entropy) relative to a random sequence (maximum entropy). Fig 3C presents a schematic of NSSC plotted against *k* for three idealized genomes of different lengths depicting the transition from full sequence space coverage to high *k*-mer specificity, passing through a minimum at k*. Example NSSC curves for eight genomes are shown in S1A Fig. The minimum NSSC and k* tend to occur where the NSSC is in the range of 0.3–0.9, most often near 0.7 (0.668 ± 0.123 [mean ± STD], n = 5805; histogram shown in S1B Fig).

For AF comparisons across all genomes, the overall optimal (minimal) *k* would ideally balance the amount of shared *k*-mers between genomes, such that closely related genomes have many in common, while phylogenetically distant genomes do not. It is also desirable that *k* be as small as possible for memory and computational considerations. From the SSC data (Fig 2A), we could already determine that *k* should be greater than 15, since some genomes contain >70% of all possible 15-mers. Even for *k* = 17, a single genome contained >15% of all possible 17-mers. We hypothesized that an optimal range of *k* for genome comparisons would occur around the *k-*mer length near the minimum NSSC. This is where *k-*mer sharing and specificity are balanced. Treating the *k-*mer length, at the minimum NSCC, as a function of the amount of total *k*-mers we extrapolate the trend from genome length to predict *k* by the size of a combined genome database. For the following computations, we restricted ourselves to one genome per genus for a total of 1634 genera representatives, as species within the same genus, and likewise strains within the same species, are known to share a very high percentage of *k*-mers. The total number of *k*-mers (duplicates included) combined for these genomes was ~2.7 x 10^11^. The sum of distinct *k*-mers from each genome, with *k*-mers occurring multiple times within a genome only counted once, is still on the order of ~2 x 10^11^ (1.92 x 10^11^ 19-mers, 2.05 x 10^11^ 21-mers, and 2.11 x 10^11^ 23-mers). We extrapolated the trend in Fig 3B by extending the line of best fit to the median genome lengths of the different colored clusters for *k* = 9–17 (linear regression: *y* = 1.97*x*−1.56). For a length of 2 x 10^11^, the estimated *k*-mer length was 21 (*y* ≈ 20.7). As expected, including genomes for all 5805 organisms would have a negligible effect on the *k*-mer length estimation as the inclusive total of distinct 21-mers was still on the order of ~2 x 10^11^. Using *k* = 21, we combined the genera representatives’ genome databases, a total of 2.05 x 10^11^ 21-mers, with the union operation (Eq 6) to obtain the set of 1.41 x 10^11^ observed distinct 21-mers. ~79% of the 21-mers were unique to a single genome, while some occurred in more than 1100 genomes stemming from highly conserved regions within the prokaryotes’ ubiquitous 16S rRNA gene. We then computed the combined NSSC for *k =* 17, 19, 21, 23. The NSCC’s were 0.97, 0.60, 0.72, 0.82 respectively for these k-mer lengths, with the minimum occurring at *k =* 19. Considering the extrapolation from the aforementioned trend and this additional NSCC analysis, we predicted *k* = 21 to be in the optimal range for comparisons across all genomes in the database because this is where there is a balance between *k*-mer sharing and specificity; It is important for the k-mer length to be at least that of the minimum NSCC (19) to have sufficient *k*-mer specificity and taxonomic resolution. At the same time, the *k-*mer length should not be too high, both for computational considerations and because the reduction of shared *k-*mers hampers the resolution of distant taxonomic relationships.

### Genome comparisons and taxonomic signals

**Fig 4:**
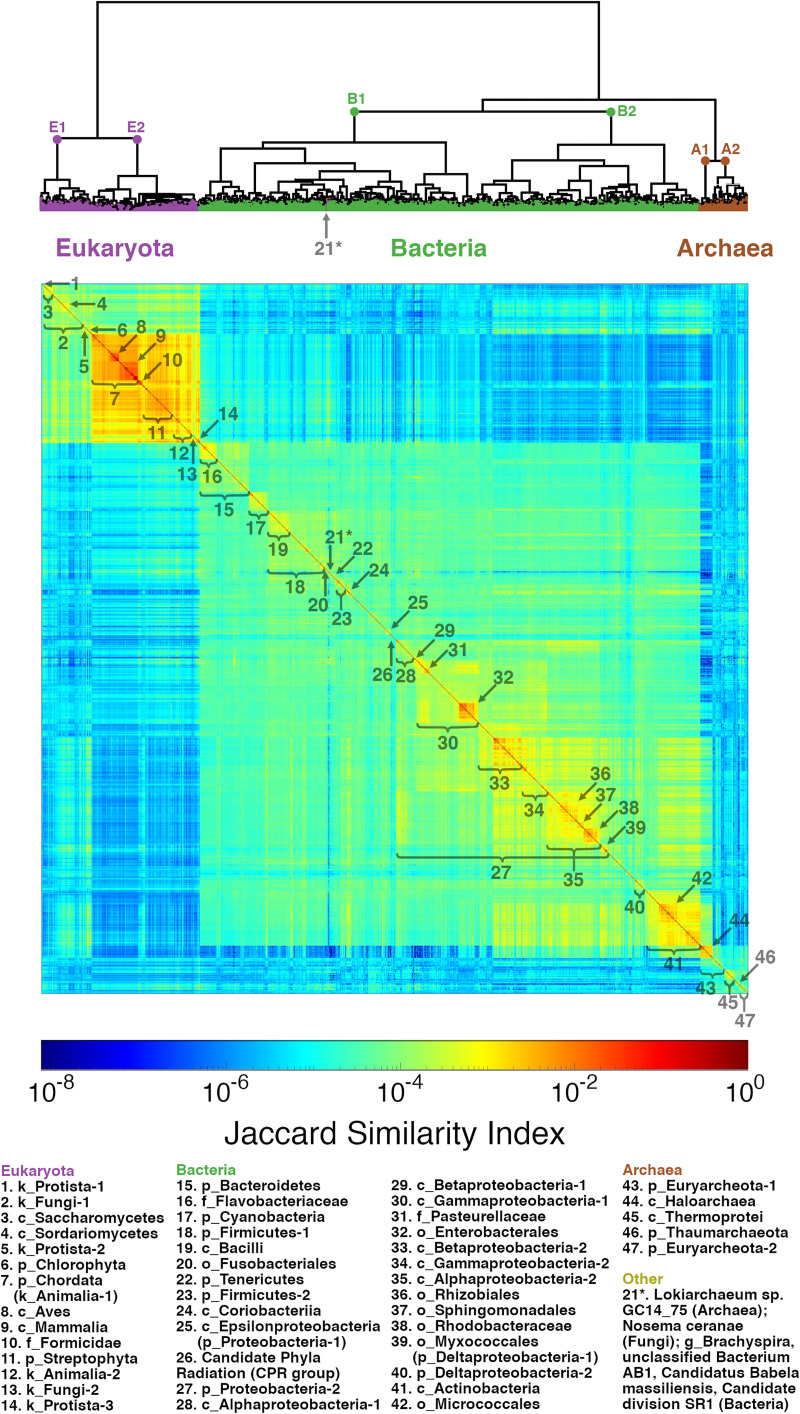
21-mer Jaccard similarity clusters genomes across different levels of taxonomy. A heatmap of pairwise 21-mer Jaccard similarity is shown for 1634 genera representatives arranged by hierarchical clustering with optimal leaf ordering to minimize the distance between successive leaves. Leaf order starts from the top left of the heatmap, and many of the clusters made up of organisms predominantly from a named taxon are numbered and labeled (brackets on the edge or arrows at a corner of clusters) with names listed in the legend (see S1 Table for ordered genera list and S2 Table for a more detailed account of named clusters). The three large clusters, corresponding to the superkingdom domain level (eukaryota, bacteria, and archaea), are colored and labeled in the hierarchical clustering tree shown above the heatmap. One group (21*; labeled in both the heatmap and tree) within the bacteria cluster is made up of a mix of an archaea, fungus, and several bacteria, all characterized by a low GC-content (<31%, bottom 5^th^ percentile). The first dichotomy of each superkingdom cluster is also labeled in the hierarchical clustering tree (E1, E2, B1, B2, A1, and A2).

Following the sequence space coverage analyses, we computed all pairwise *k*-mer set comparisons (Fig 1B) for the 1634 genera representatives with *k* = 11 and 21. Once the boundary of the minimum NSCC is passed, we do not expect very large differences when comparing adjacent *k*-mer lengths (i.e. 19 vs. 21), because we assume there to be only a slight tradeoff between *k-*mer sharing and specificity. For that reason and due to computational constraints, we performed the following analyses at k-mer lengths in increments of 10. However, for larger *k*-mer lengths, the computation time and memory requirements were still prohibitive for eukaryotic genome comparisons. Thus, for *k* = 31 and 41 we limited ourselves to pairwise comparisons for all 1266 prokaryotic genera representatives. Specifically, for every pair of genomes we first computed the union of their *k*-mer sets and then calculated the Jaccard similarity (Eq 1) to form a pairwise similarity matrix (Fig 1C). From this matrix, a recapitulated phylogenetic/taxonomic tree was generated by hierarchical clustering (Fig 1D) with optimal leaf ordering to minimize the distance between neighboring leaves. A heatmap of the 21-mer pairwise similarity matrix for all genera is presented in Fig 4 with the tree shown above; an unrooted view of the tree is also displayed in S2A Fig. While the relations between these clusters have higher dimensionality than could be presented in the ordered heatmaps, they are well-suited for visualizing large-scale comparisons. Some of these relationships piqued interest for further in-depth investigation (outside the scope of this work) and are described in the following paragraphs.

For *k* = 21, the three superkingdoms form three distinct clusters with the exception of one group within the bacteria cluster (Fig 4, group 21*), which contains the archaea *Lokiarchaeum* sp. GC14_75, microsporidian fungus *Nosema ceranae*, bacterial genus *Brachyspira*, and several candidate/unclassified bacteria. Genomes in this anomalous group are of highly variable length (~0.5-8Mbp), but all have low GC-content (<31%, in the bottom 5th percentile).

Eukaryota very clearly split into two main clusters in the heatmap (Fig 4, E1 and E2), which largely correspond to a threshold of genome lengths at ~100Mbp, or the majority of fungi vs. plants and animals. Protists, which are made up of a diverse group defined as eukaryotes not belonging to fungi, plants or animals, are accordingly split far apart from each other into three separate groups (1, 5, 14). The first main cluster of eukaryotes (E1), with lower genome lengths, contains the plant phyla Chlorophyta (group 6; green algae) and Rhodophyta (red algae). Most green algae (8/9) are clustered together and ordered closest to the second large eukaryotic cluster of plants and animals. However, red algae, for which there were only three genera representatives, are split within protist clusters (genus *Galdieria* in group 1 and genera *Cyanidioschyzon* and *Chondrus* in group 5). Animals are separated into groups containing chordates, ants (Formicidae family) and non-chordates (groups 7, 10, and 12 respectively) with the land plants, phylum Streptophyta (group 11), ordered between ants and non-chordates. Interestingly, 4 of 5 nematode genera (phylum Nematoda, see S1 Table), genera *Tetranychus*, *Dermatophagoides*, and *Pediculus* from phylum Arthropoda, and several fungi and protists (groups 13, 14) are clustered together, within the second main cluster of eukaryotes (E2), and placed closest to the bacterial phylum Bacteroidetes (group 15), specifically neighboring the family Flavobacteriaceae (group 16).

**Fig 5:**
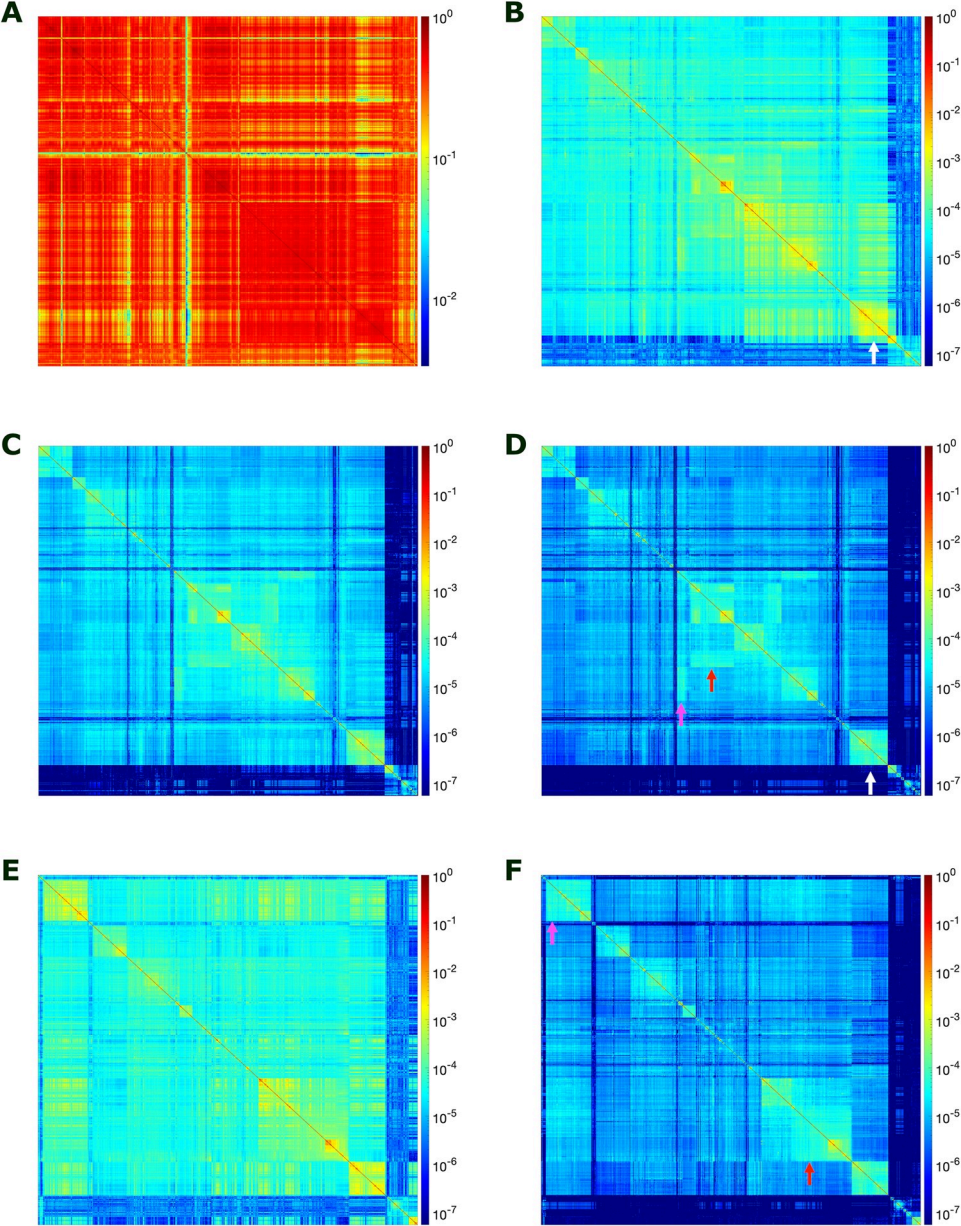
Prokaryotic genome clustering differs significantly with varying *k*-mer lengths used to compute similarity. (A-D) Heatmaps of pairwise 11-, 21-, 31-, and 41-mer Jaccard similarity are shown for 1266 prokaryotic genera representatives arranged by hierarchical clustering with optimal leaf ordering by 21-mer similarity (same as in Fig 4). (E-F) For comparison, heatmaps of pairwise 21- and 41-mer Jaccard similarity are shown ordered by optimal leaf ordering by 41-mer similarity. For increasing *k*-mer lengths, the signal of similarity between some groups is diminished, for example between Haloarchaea and a group of bacteria that likely share horizontally transferred genes (B and D white arrows). Conversely, the signal of similarity between some split taxons becomes more apparent off the diagonal with increasing *k*, for example Alphaproteobacteria (D and F, magenta arrows) and Gammaproteobacteria (D and F, red arrows). While more of the genomes in these proteobacteria classes are within a single group, the groups are separated further apart from each other with 41-mer ordering as this phylum-level signal is diminished (F). Color legend for Jaccard similarity is shown to the right of each plot.

**Fig 6:**
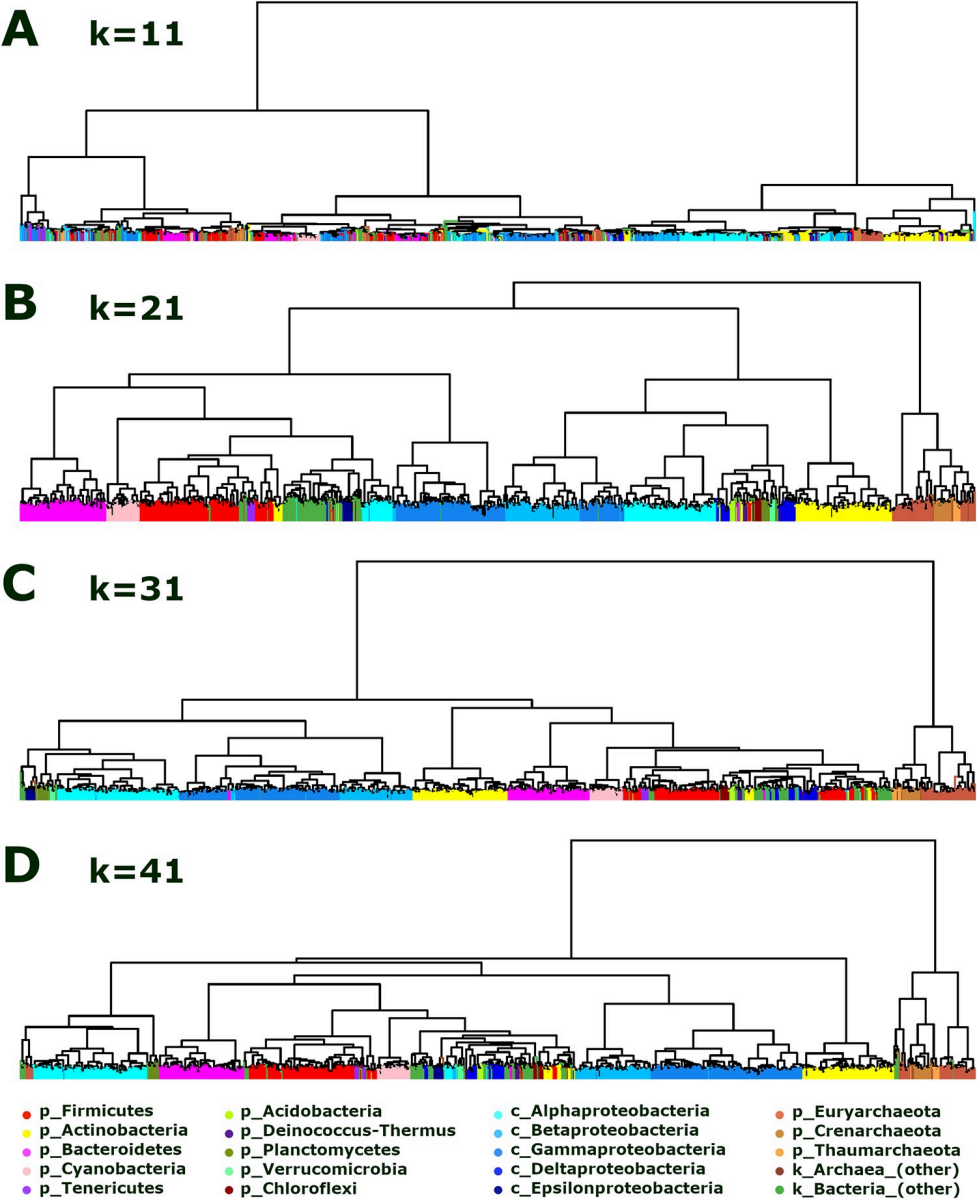
Hierarchically clustered trees of large prokaryotic taxons visually demonstrate an optimal range of *k*-mer lengths for genome comparisons. (A-D) Hierarchical clustering trees with optimal leaf ordering computed from pairwise 11-, 21-, 31-, and 41-mer Jaccard similarity of 1266 prokaryotic genera representative genomes. Leaves are colored by large taxonomic groups, including 11 bacterial phyla and 3 archaeal phyla, with proteobacterial classes separated (see legend at bottom). For short *k*-mer lengths, genomes do not cluster well by taxon groups due to *k-*mer homoplasy as seen by the 11-mer tree with mixed leaf colors (A). For large *k*-mer lengths, the similarity for distant taxons reduces until the signal is too low to cluster them together (few if any long *k*-mers shared), as is seen by the 41-mer tree which has more bacteria and archaea mixed together than the 21- and 31-mer trees (see left- and right-end groups in D). The 21- and 31-mer trees (B and C) separate bacteria and archaea well and cluster phyla together closely. The 31-mer tree clusters Alpha and Gammaproteobacteria (light blues) together better than the 21-mer tree, but Delta and Epsilonproteobacteria are further away (dark blues). These *k*-mer lengths fall in an optimal range which balances *k*-mer sharing and specificity.

While bacteria and archaea tend to cluster well within phyla for *k* = 21, the phylum Proteobacteria (Fig 4, group 27) was fragmented by class and even split by the first division of bacteria (Fig 4, B1 and B2). Heatmaps and trees for prokaryotic genome comparisons with *k*-mer lengths 11, 21, 31, and 41 are shown in Figs 5 and 6, respectively. To facilitate comparisons, heatmaps in Fig 5A–5D are ordered by the 21-mer tree, and in Fig 5E and 5F the 21- and 41-mer heatmaps are ordered by the 41-mer tree. For *k* = 11, many *k*-mers are covered and shared, even between distant genomes, leading to low resolution and highly heterogenous clusters (Figs 5A and 6A). In contrast, for *k =* 21 and 31, most genomes clustered well within named taxons across all levels from family to superkingdom (see Figs 4, 5B, 5C and 6B–5C and S1 and S2 Tables). For *k* = 41, *k-*mers are very specific leading to low signal for more distant genomes and many taxonomic groups are mixed even between archaea and bacteria (Fig 6D). Proteobacteria were not all clustered together for any of the *k*-mer lengths (Fig 6). The majority of genomes in Alpha and Gammaproteobacteria classes clustered together better for *k* = 31 than *k* = 21 (see tree leaves colored by shades of blue in Fig 6). For higher *k*-mer lengths, the similarity between these split proteobacteria classes can be seen clearly as off-diagonal signals in the heatmap (see red and magenta arrows in Fig 5). However, for *k* = 41 the signal dampens and groups of Alphaproteobacteria are separated (cyan Fig 6D). A full list of genera representatives ordered by 21-mer hierarchical clustering with optimal leaf ordering is provided in the supplemental information (S1 Table).

At the bacteria-archaea border for *k* = 21 the classes Actinobacteria and Haloarchaea (Fig 4, groups 41 and 44/A1) neighbor each other with relatively high similarity (~10^−4^). This relationship is greatly diminished, and eventually lost, for higher lengths of *k* (see white arrows in Fig 5B and 5D). Unlike other Euryarcheota, Haloarchaea have significantly higher Jaccard similarity to the second main bacterial cluster (B2), median = 4.2 x 10^−5^ (IQR = 1.6–8.2 x 10^−5^; n = 14877 pairs), than the first (B1) cluster, median = 1.9 x 10^−6^ (IQR = 0.9–4.5 x 10^-6^; n = 18647 pairs), as can be seen in the heatmap. Haloarchaea and this second main bacterial cluster, which includes Actinobacteria, a large group of Proteobacteria, and the phyla described above cluster with Deltaproteobacteria, both have higher relative similarity to a portion of the first main cluster of eukaryotes including the green algae, protists (group 5), and many fungi (group 2), excluding Saccharomycetes (group 3).

Given that the prokaryotic tree was recapitulated well by both 21- and 31-mer based clustering and that 21-mer comparisons were more efficient, we proceeded to focus on 21-mers for the following analyses. For a large-scale visualization of the comparison between the reference phylogenetic tree and the hierarchical clustering tree, based on 21-mer Jaccard similarity for genera representatives, we plotted both side-by-side in a tanglegram (S2B Fig). The reference tree contains many polytomies, or nodes with more than two branches, *e*.*g*. a single phylum may contain several classes, which complicates performing a direct one-to-one tree comparison. Still, it is evident from the leaf ordering that many large subtrees are indeed comparable from our tree to the reference (large bands of linking lines in S2B Fig), as was to be expected from the analysis of the heatmap (detailed above). The sole anomalous group (Fig 4, group 21*) is also clearly visible with the linking lines colored by superkingdom domain. When comparing only the Mammalian class (S3 Fig), the reference tree can be fully resolved by intermediate taxon labels and indeed most of the subtrees are identical or near-identical in topology (shown in different colors in S3 Fig). The most notable disagreement between the clustered Mammalian tree and the reference is for the treeshrew genus, *Tupaia*, whose linking line intersects most lines. However, as noted in the Methods, *Tupaia*’s placement has been debated in the literature recently [79, 80], in favor of placement as a sister clade to Glires (rodents and lagomorphs), consistent with our tree. We calculated the generalized Robinson-Foulds distances from the NCBI reference to our hierarchically clustered trees. To put the distances in context we compared 100 pairs of random trees with the same number of leaves. For the mammalian subtree, the distance from the reference was 0.536 compared to 0.852±0.018 (mean ± std) for random trees with 44 leaves, and for the full tree, the distance was 0.655 compared to 0.917±0.001 (mean ± std) for 100 random trees with 1624 leaves. Overall, the 21-mer based genome comparisons capture much of the taxonomical information from the reference database, as evidenced by the heatmap and tanglegram analyses.

**Fig 7:**
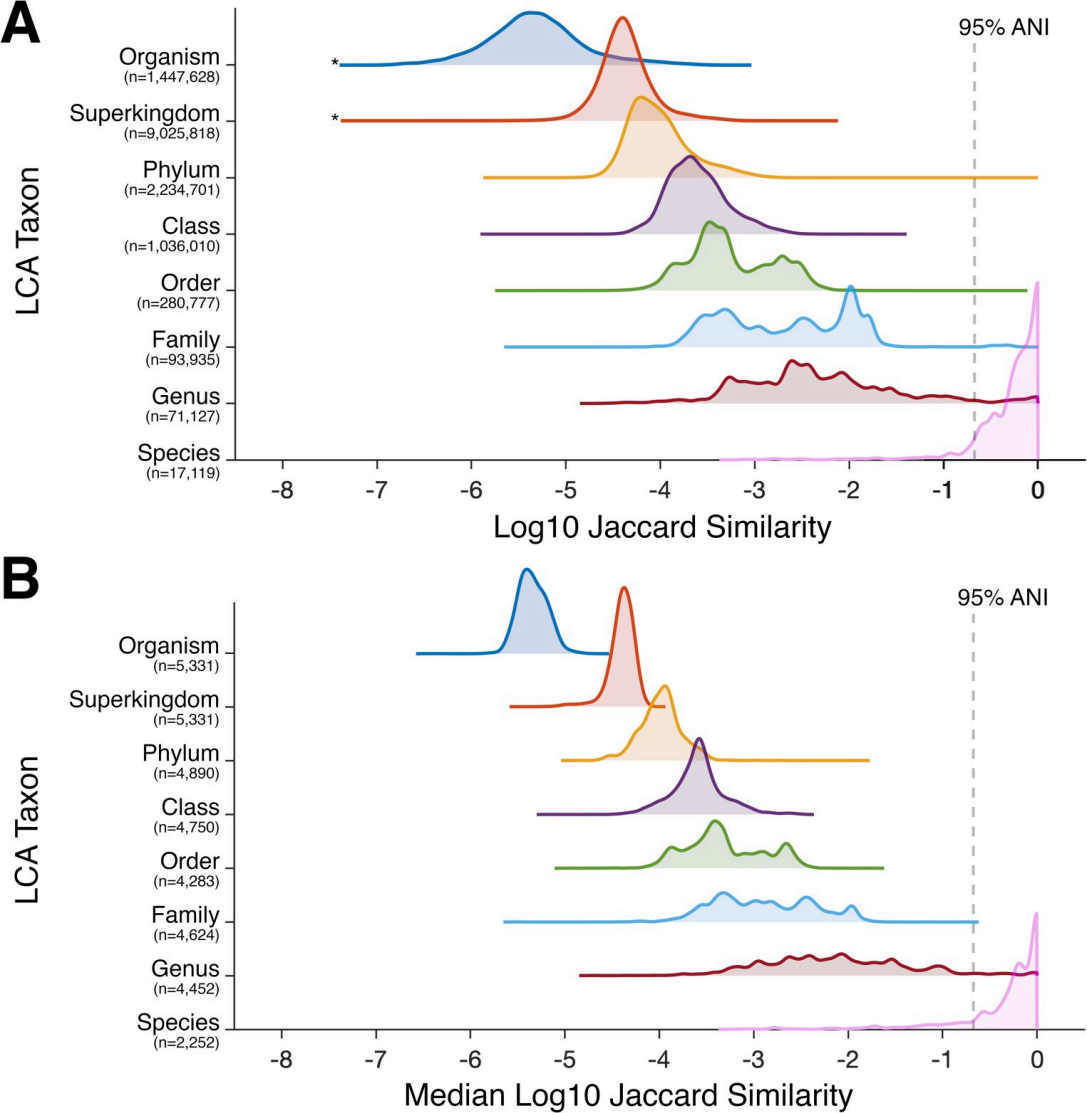
Distributions of log-transformed Jaccard similarity for different lowest-common-ancestor (LCA) prokaryotic taxon levels. Ridgeline plots show the distributions of log10 Jaccard similarity (A) and median log10 Jaccard similarity (B) at different LCA taxon levels. Distributions were computed by kernel density estimation (see Methods). The dotted lines represent an estimated 95% average nucleotide identity (ANI), at approximately -0.67 log10 Jaccard similarity (see Eq 2), which is commonly used as a species-level threshold. Asterisks adjacent to the organism and superkingdom distributions in **A** represent the 1177 out of ~1.3M pairs which shared zero *k*-mers; 252 of these pairs with LCA above the phylum level, *i*.*e*. bacteria-archaea pairs, and 925 archaea-archaea pairs.

Lastly, we turned our attention to analyzing similarity at different levels of taxonomy. For every pair of the 5331 prokaryotic genomes compared (~14.2M comparisons, comprising 3517 species), the similarity score was classified by the lowest-common-ancestor (LCA) taxon level of the pair. Distributions of the log-transformed, as well as median log-transformed, Jaccard similarities for *k* = 21, are presented for each of the major taxon levels (Fig 7). Overall, the similarity scores increased for lower (closer) LCA taxon levels, as expected. ~91.0% of comparisons within the species level had an estimated ANI above the widely accepted 95% species-level threshold (Eq 2). Although 102 species had at least one pair of strains with an estimated ANI below 95% (S3 Table), 50 of these species had at least one strain-pair below 90% estimated ANI, which are very likely indicative of misclassifications. On the other hand, 22 species pairs from different genera had an estimated ANI above the 95% species threshold (S4 Table), in addition to all pairs between *Escherichia* and *Shigella* which were placed in separate genera based on medical relevance [83]. These are also very likely indicative of misclassifications. 544 species pairs from the same genus with at least one pair of strains having ANI above 95% are provided in S5 Table. At the LCA levels of class and above, the distributions were unimodal and were narrower for median similarities (Fig 7B). However, at the genus, family, and order levels, distributions were multimodal with relatively wide overlapping regions. These data suggest that, unlike for species, there may not be practical universal thresholds at the taxon levels of genus, family, and order. Correspondingly, on the basis of these similarity scores alone, it would be difficult to accurately predict the degree of taxonomic relatedness for two organisms that are phylogenetically in the same phylum, yet are more distant than strains of the same species.

**Fig 8:**
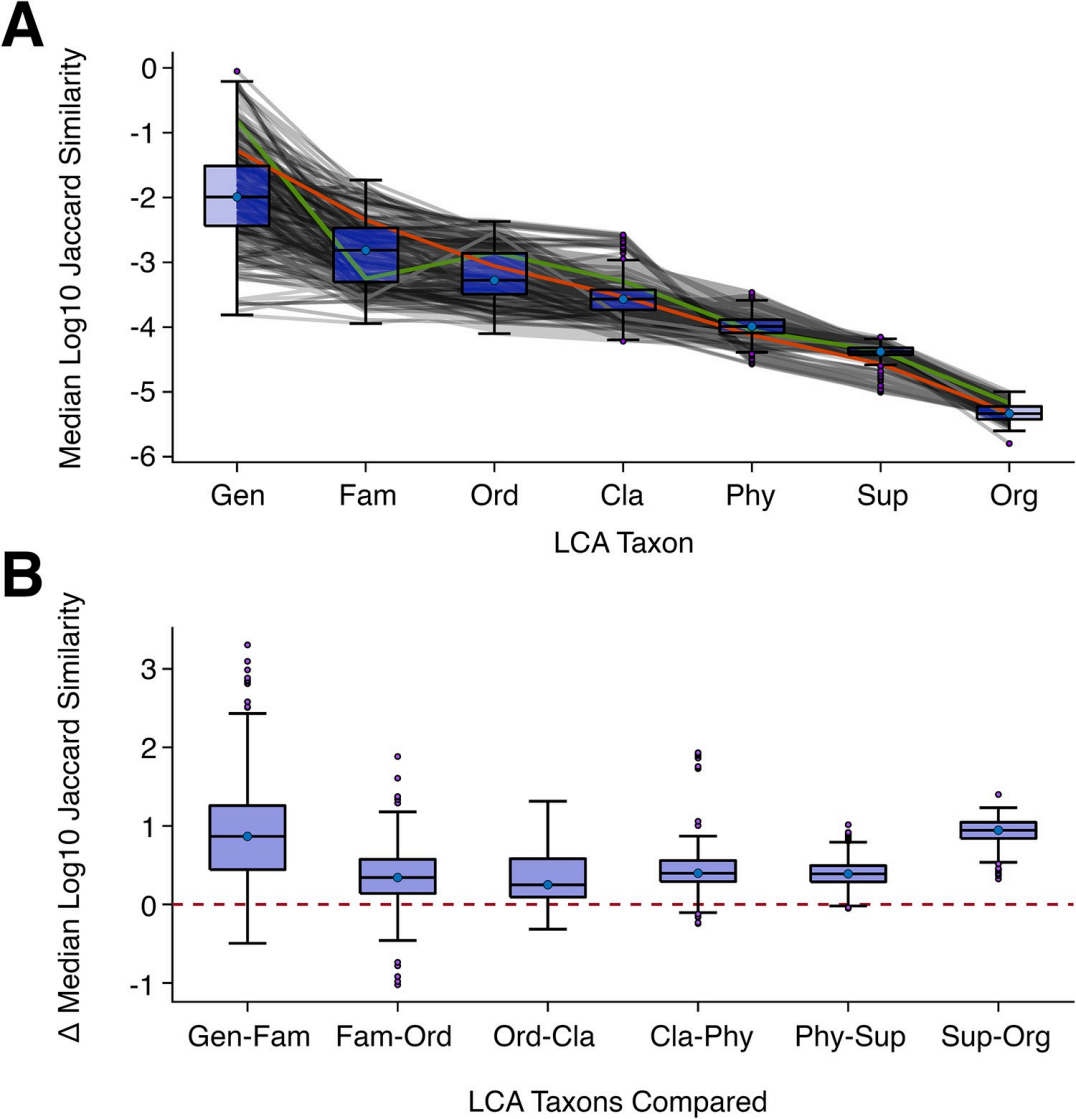
Aberrant trajectories of median similarity across taxonomic levels indicate potential misclassifications in prokaryotic reference databases. (A) For prokaryotic genera representatives that had at least one pairwise comparison at all lowest-common-ancestor (LCA) levels from genus to cellular organism (n = 291), we plotted trajectories of their median log10 Jaccard similarity for *k* = 21 (gray lines), along with overlaid boxplots to show the overall distribution at each level. Two trajectories are highlighted to show an example of median similarity always decreasing as LCA distance increases (orange; *Bradyrhizobium diazoefficiens*) and an example of an aberrant trajectory for which median similarity increases as LCA taxon goes from family to order (green; *Roseomonas gilardii*). (B) For the same group of prokaryotes, boxplots show the distribution of delta median log10 Jaccard similarity for increasing taxon level pairs (*e*.*g*. genus *minus* family, family *minus* order, *etc*). The horizontal red dotted line represents an equivalent median similarity from a genome to the two compared LCA taxons. Negative values, below this line, are unexpected and are potentially due to misclassifications in the database, *e*.*g*. a species having a higher median similarity to organisms which share the same order than to those in its family (green line in A). In total, about one third (106/291) of the genera analyzed had at least one value below zero.

Although distributions of similarity scores were multimodal, wide, and overlapping for some taxon levels, we expected that for a single genome the average similarity scores would decrease when moving to higher (more distant) LCA taxon levels (see Eq 9, Methods). To evaluate this hypothesis, we plotted the trajectories of median log10 Jaccard similarity (*k* = 21) for the 291 prokaryotic genera representatives that had at least one pairwise comparison at all LCA levels from genus to cellular organism (Fig 8A). Approximately two-thirds of these representatives had decreasing similarity values, obeying Eq 9. The remaining, however, had an aberrant trajectory where at least one taxon level had a median similarity value higher than that of a closer taxon level. We also plotted the distribution (boxplots) of the delta in median log10 Jaccard similarity for increasing taxon level pairs (*e*.*g*. genus vs family, family vs order, *etc*.; Fig 8B), such that values below zero would potentially indicate misclassifications in the database, either in the placement of a species or in the placement of its relatives. Overall, ~93% of the values for the delta median similarity at the taxon pairs analyzed were above zero. In addition, we provide a table of the counts where the delta in median (and max) log10 Jaccard similarity between different taxon levels was below multiple thresholds (0, -0.05, -0.1, -0.5) for all 1266 prokaryotic genera representatives (S6 Table). Similar to the analysis restricted to the 291 genera representatives with relatives at each LCA taxon level (Fig 8), approximately one-third had at least one delta median similarity value below zero. In comparison, many more (>60%) had a delta maximum similarity value below zero. This is because the maximum is very sensitive measure, *e*.*g*. a single relative can change the maximum similarity value for many of its relatives at different LCA taxon levels without having any effect on their median similarity values. In some cases, these apparent violations of taxonomic similarity may be due to biases in our database or in the measurement of similarity, for example low similarity when comparing relatives with widely varying genome lengths. Nonetheless, many of the violations are likely to be due to misclassifications in the database, a result that warrants further investigation and has implications for a wide range of downstream bioinformatics processes dependent on the fidelity of these data.

## Discussion

In this work, we characterized several global, *k*-mer based, informational properties of complete genomes from a large curated database. Our sequence space coverage (SSC) analysis was consistent with the findings of Liu *et al*. in that SSC largely depends on genome length and GC-content [14]. Maintaining a GC-content significantly far from 0.5 favors the presence of some *k*-mers over others and thus reduces the sequence’s expected entropy and SSC. As shown, genome length was a much larger contributor towards SSC and so we expanded on our analysis by normalizing for this factor, through the evaluation of random sequences of similar lengths, in what is to our knowledge the first analysis of normalized sequence space coverage (NSSC). The insights on *k*-mer specificity gleaned from the NSSC and combined SSC analysis of thousands of genomes led us to determine that 21 and 31 are within the optimal range of *k*-mer lengths for large-scale, alignment-free (AF), whole-genome comparisons across higher domains. This result is consistent with the work of Fan *et al*. [46] towards optimizing parameter selection for AF techniques, based on the statistical properties of the pairwise distances between tens of eukaryotic genomes, and the empirically derived default (*k* = 21) for the ubiquitously used Mash software [44].

In practice, *k*-mer lengths within a small range are likely to yield near equivalent results, thus we checked values of k from 11 to 41 with intervals of 10. The optimal choice of *k*-mer length mainly depends on the data/database being used. It is critical that the *k*-mer length be large enough to accommodate the quantity and diversity of *k*-mers in the database such that distantly related genomes share few, but some, *k*-mers (likely from homologous regions) and closely related genomes share many, but not all, *k*-mers. Since the choice of optimal *k*-mer length depends on the log-scale genome lengths, the database size would have to increase by an order of magnitude to change the estimate of the value that produces the minimum NSCC from 21 to 23. For analyses restricted to prokaryotic genomes, however, a lower *k*-mer length, such as 17–19 (but not much lower), would be sufficient and possibly more efficient for large-scale comparisons. Accordingly, FastANI uses *k* = 16 by default [43]. On the other hand, for optimal strain-level resolution, higher *k*-mer lengths are needed, even *k* ≥ 50. These are often referred to as genome-specific markers [84, 85]. In the context of genome classification, genome-specific markers are most efficient if there is a high-quality comprehensive reference database, and once a close match to a related species is obtained the rest of the taxonomy is already known. This, however, is not always the case. Therefore, approaches utilizing multiple different *k*-mer lengths, such as MetaPalette [47], are of interest towards extracting high resolution signals of genome comparison scores across all taxonomic levels.

Beyond characterizing the informational properties of genomes, we showed that taxonomic relationships are well-captured by hierarchical clustering of 21-mer based pairwise similarity scores. While the aim of this work was to perform a broad large-scale analysis, several specific taxonomic relationships stood out as noteworthy. For example, *Lokiarchaeum*, one of the only genera not clustered within its superkingdom level (see Fig 4, group 21*), belongs to the Asgard superphylum (of archaea), which are thought to be the closest prokaryotic relatives of eukaryotes [86–89]. The classification of Rhodophyta (red algae) above the level of order is still debated, specifically whether it should be classified within Plantae (along with Viridiplantae) or as part of the kingdom Protista [90, 91]. Our *k*-mer-based analysis suggests the latter. Lastly, in regards to the observation that Haloarchaea had significantly higher similarity to a large group of bacteria for *k* = 21, Nelson-Sathi *et al*. have proposed that a massive horizontal gene transfer of over 1,000 eubacterial genes transformed a methanogenic recipient into the haloarchaeal ancestor [92]. We emphasize that this analysis did not include any direct computations of alignment or alignment-fractions, solely exact *k*-mer matches by set comparisons. More in-depth investigations, such as into the identity of specific *k*-mers that are shared between taxons and what genes they derive from, were outside the scope of this work.

As previously noted, the KEGG GENOME database [52] was chosen because it is one of the few curated databases for complete genomes. Other databases, such as those of NCBI are known to include misassembled, incomplete, contaminated, and/or misclassified genome sequences [93–96]. Still, we detected many potential errors in the KEGG database through our efforts to uncover the overall consistency of the taxonomic relationships from our AF approach to the reference database and by analyzing each lowest-common-ancestor (LCA) taxon level separately. The reference NCBI Taxonomy database combines both phylogenetic and taxonomic knowledge from a diverse collection of sources, and it provides a disclaimer that it is not a taxonomic authority. Despite its limitations, including polytomies and missing/unclassified/misclassified taxon levels, it is still ubiquitously used as a reference and includes both eukaryotic and prokaryotic organisms. The Genome Taxonomy Database (GTDB), very recently released by Parks *et al*., aims to provide a comprehensive standardized prokaryotic taxonomy utilizing similar AF techniques [49, 50]. Remarkably, in their database more than half of the >90k genomes, including both complete and incomplete genomes, had changes to their existing taxonomy from NCBI, including most of the potential errors detected in this work.

In addition to the issue of flawed genome sequences in the reference database, there are many challenges and limitations in AF analyses. One major challenge is sampling bias in the database. Many of the presently known sequenced genomes are specifically of interest to human health and/or culturable, *e*.*g*. *Escherichia coli* is represented by 65 different strains in the KEGG database. We attempted to mitigate this bias by considering only one representative per genus in many of our analyses. In addition, rapid evolutionary divergence of a group of taxons from a common ancestor can make resolving all branches of the phylogenetic tree very challenging to near impossible [97]. Horizontal gene transfer may also present difficulties as it may increase the similarity of phylogenetically distant species [98]. On the other hand, massive gene loss, as is the case for many endosymbiotic bacteria, will decrease the similarity of phylogenetically close species [99]. Indeed, in the KEGG database, several endosymbionts led to some of the violations in similarity scores observed by taxon level.

At the core of these difficulties is the subtle tension between phylogeny and taxonomy, *i*.*e*. should classifications be firmly based on evolutionary relationships (marker genes like 16S) or on other practical shared characteristics (genotype or phenotype)? Some questions do not have clear answers, for example: at what point should the classification of an endosymbiont that has lost major parts of its genome be changed, if at all? How should we treat historical classifications based on limited, erroneous, and/or inconsistent observations or on explicit exceptions due to medical relevance, such as the separation of *Escherichia* and *Shigella* to two separate genera while their average nucleotide identity is above the species threshold. It is important to consider the utility of the classifications themselves while grappling between the significance placed on evolution, genotype, or phenotype. The importance of reference databases for bioinformatic applications cannot be understated. Diversity analysis, core and pan-genome analysis, and genome assembly assessment all depend on the fidelity of taxonomical and genomic information present in the references. With the explosion of high throughput genome sequencing of unculturable microbes, we advocate for the move towards standardized, quantitative genotypic classifications, such as that available from the GTDB [49, 50], with automated error correction. Undoubtedly, alignment-free approaches will provide further improvements throughout the field of bioinformatics, such as in microbial identification and metagenomic assembly/binning.

## Supporting information

S1 Fig Examples of normalized sequence space coverage (NSSC) curves and the distribution of minimum NSSC. (A) NSSC is plotted against *k*-mer length for eight genomes as an example: *Homo sapiens*, *Gallus gallus*, *Dinoponera quadriceps*, *Theobroma cacao*, *Saccharomyces cerevisiae*, *Escherichia coli*, *Sorangium cellulosum*, and *Nanoarchaeum equitans* (see Fig 3C for a schematic of idealized NSSC curves). Genome lengths varied widely, from ~0.5Mbp for *N*. *equitans* to ~3Gbp for *H*.*sapiens*. A histogram of the minimum NSSC for all 5805 genomes is shown in (B). The minimum NSSC usually occurs within the range 0.4–0.9 (0.668 ± 0.123 [mean ± STD]).
(PDF)

S2 Fig Large-scale visualization of hierarchical clustering versus reference phylogenetic tree shows many comparable large subtrees. (A) An unrooted view of the recapitulated phylogenetic/taxonomic tree by hierarchical clustering, comparable to the phylogram view shown below in panel B and at the top of Fig 4, is shown with leaf edges colored by superkingdom domain (eukaryota, bacteria, archaea). (B) The 21-mer hierarchical clustering tree is compared with the reference phylogenetic tree. Lines in the tanglegram plot are colored by superkingdom domain as in panel A. Unlike the hierarchical clustering tree, the reference phylogenetic tree contains many polytomies, or nodes with more than two branches, and evaluating their similarity is challenging. However, it is suitable to visualize that many large subtrees from the clustering are comparable to the reference, indicated by large bands of lines, as well as to detect any anomalous groups, *e*.*g*. the purple and gold lines intersecting green lines (only group 21* from Fig 4).
(PDF)

S3 Fig Mammalian order and family subtree topology derived from hierarchical clustering is very similar compared to the reference phylogenetic tree. The Mammalian class subtree (left) from the hierarchical clustering of 1634 genera representatives by 21-mer Jaccard similarity (Fig 4, group 9) and the corresponding reference phylogenetic tree (right) were plotted in a tanglegram. Polytomies in the reference phylogenetic tree were fully resolved using intermediate taxon labels from the database, and in few cases from the literature (see Methods for further details). Identical subtrees and their linking lines are colored (different colors). Non-identical branches/leaves and their linking lines are shown in black. Most subtrees, representing order and family phylogeny, were identically represented in the hierarchical clustering and some branches only had very minor differences, for example the connection between *Pongo* and *Nomascus* genera within the Hominoidea superfamily.
(PDF)

S1 Table Genera representative list ordered by hierarchical clustering with optimal leaf ordering. Superkingdom, phylum, and genus is listed for all 1634 genera representatives in the order of the optimal leaf ordering from the 21-mer Jaccard similarity hierarchical clustering (see Methods). The numbering starts from the top left corner of the heatmap in Fig 4 and from the top in the left tree of S2B Fig.
(XLSX)

S2 Table Clusters derived from hierarchical clustering of 21-mer Jaccard similarity are predominantly composed of named taxons. Clusters which have a majority of genera representatives from the same taxon are labeled with the corresponding taxon name (column 3) and taxon level (column 4). The number of genera that belong to the taxon and are in the cluster is given (column 5), as well as the total size of the cluster (column 6), the genus range (column 2; from S1 Table), and the total genera (out of 1634 representatives) that belong to the taxon. Named clusters, readily visualized in the heatmap of Fig 4, were labelled and numbered with a Heatmap/group ID (column 1). If multiple clusters from different subtrees (top of Fig 4) have the same taxon name, then a number is added to the name to disambiguate, unless these clusters are neighboring in the ordered list, as is the case for Proteobacteria-2 (Heatmap ID/group 27).
(XLSX)

S3 Table Prokaryotic species containing ≥1 strain-pair with estimated average nucleotide identity (ANI) below species-level threshold. Column 1 lists the names of 50 prokaryotic species where the minimum strain-pair estimated ANI was less than 90%. Strain-pairs below this threshold are very likely misclassified as being the same species. Column 2 lists the names of 52 prokaryotic species where the minimum strain-pair ANI was between 90–95%.
(XLSX)

S4 Table Prokaryotic species pairs in different genera with estimated average nucleotide identity (ANI) above species-level threshold. Columns 1–2 show pairs of prokaryotes in different genera whose estimated ANI (column 3) is higher than 95%. These are very likely misclassified as being in different genera. As expected, all species pairs within *Escherichia* and *Shigella* (represented in the last row) have estimated ANI above this threshold, as they were classified to different genera for medical relevance.
(XLSX)

S5 Table Prokaryotic species pairs in the same genera with estimated average nucleotide identity (ANI) above species-level threshold. Columns 1–2 show pairs of prokaryotic species within the same genera that have at least one pair of strains whose estimated ANI is above 95%, the commonly accepted species-level threshold. These species pairs should likely be investigated further to determine if there are strains misplaced between them and/or if they should be merged to one species.
(XLSX)

S6 Table Counts of prokaryotes where the delta in similarity across compared taxonomic levels is below thresholds indicating potential misclassification. For all prokaryote genera representatives (n = 1266), we computed the median (and max) log10 Jaccard similarity for every lowest-common-ancestor (LCA) taxon level for which there was at least one pairwise comparison. We then computed the delta between different taxon levels, shown in the leftmost column (*e*.*g*. species vs genus, species vs family, *etc*.), and present the number of genera that had at least one comparison below different thresholds (column headers).
(XLSX)
