# Supplementary material for: Large-scale k-mer-based analysis of the informational properties of genomes, comparative genomics and taxonomy
Source: PLoS One. 2021 Oct 14;16(10):e0258693. doi: 10.1371/journal.pone.0258693 (PMC8516232; doi:10.1371/journal.pone.0258693)
Supplement: S1 Fig — (A) NSSC is plotted against k-mer length for eight genomes as an example: Homo sapiens, Gallus gallus, Dinoponera quadriceps, Theobroma cacao, Saccharomyces cerevisiae, Escherichia coli, Sorangium cellulosum, and Nanoarchaeum equitans (see Fig 3C for a schematic of idealized NSSC curves). Genome lengths varied widely, from ~0.5Mbp for N. equitans to ~3Gbp for H.sapiens. A histogram of the minimum NSSC for all 5805 genomes is shown in (B). The minimum NSSC usually occurs within the range 0.4–0.9 (0.668 ± 0.123 [mean ± STD]). (PDF) [file pone.0258693.s001.pdf]

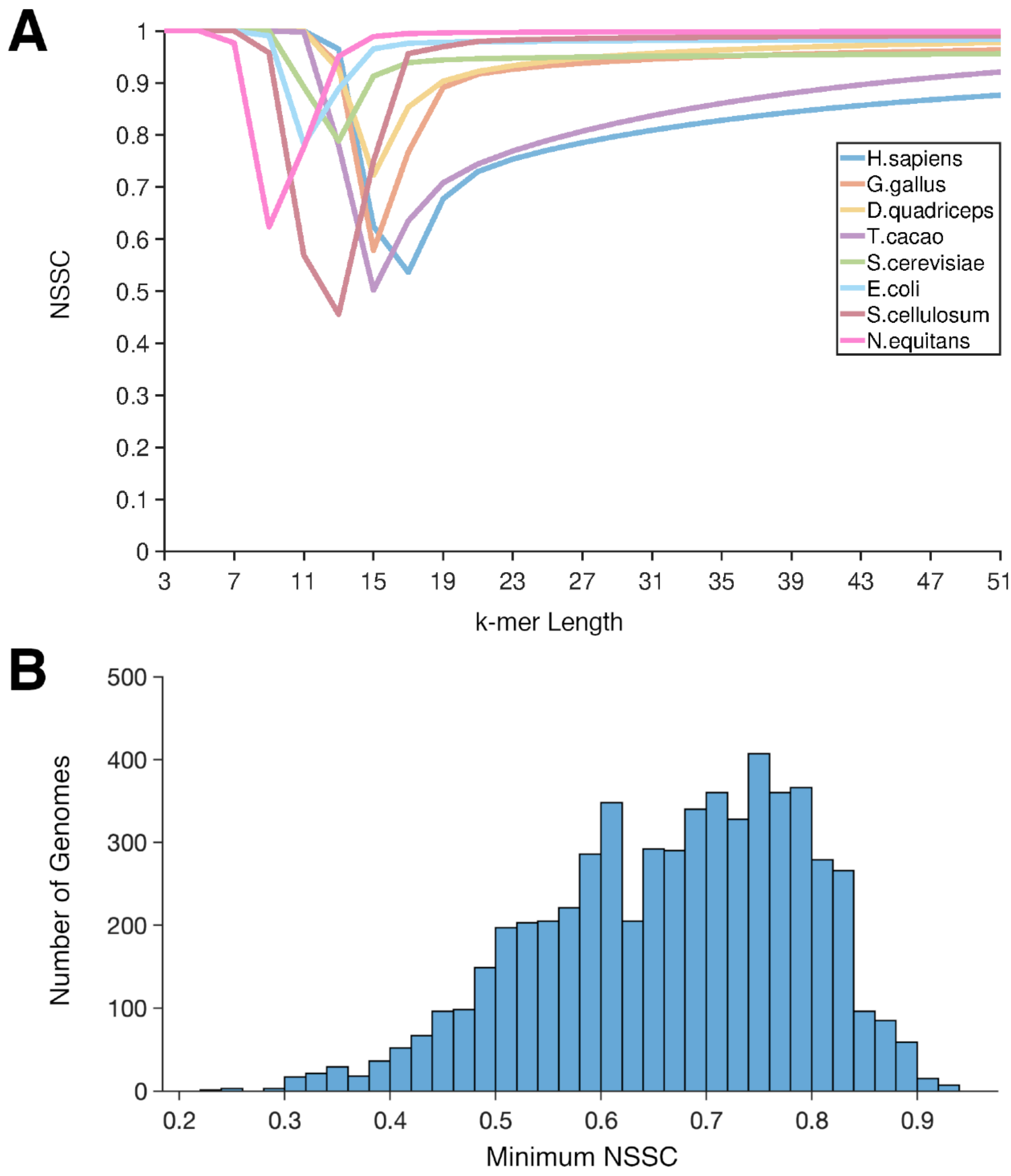

S1 Fig. Normalized sequence space coverage (NSSC) curve examples and the distribution of minimum NSSC.
