# Supplementary material for: Large-scale k-mer-based analysis of the informational properties of genomes, comparative genomics and taxonomy
Source: PLoS One. 2021 Oct 14;16(10):e0258693. doi: 10.1371/journal.pone.0258693 (PMC8516232; doi:10.1371/journal.pone.0258693)
Supplement: S2 Fig — (A) An unrooted view of the recapitulated phylogenetic/taxonomic tree by hierarchical clustering, comparable to the phylogram view shown below in panel B and at the top of Fig 4, is shown with leaf edges colored by superkingdom domain (eukaryota, bacteria, archaea). (B) The 21-mer hierarchical clustering tree is compared with the reference phylogenetic tree. Lines in the tanglegram plot are colored by superkingdom domain as in panel A. Unlike the hierarchical clustering tree, the reference phylogenetic tree contains many polytomies, or nodes with more than two branches, and evaluating their similarity is challenging. However, it is suitable to visualize that many large subtrees from the clustering are comparable to the reference, indicated by large bands of lines, as well as to detect any anomalous groups, e.g. the purple and gold lines intersecting green lines (only group 21* from Fig 4). (PDF) [file pone.0258693.s002.pdf]

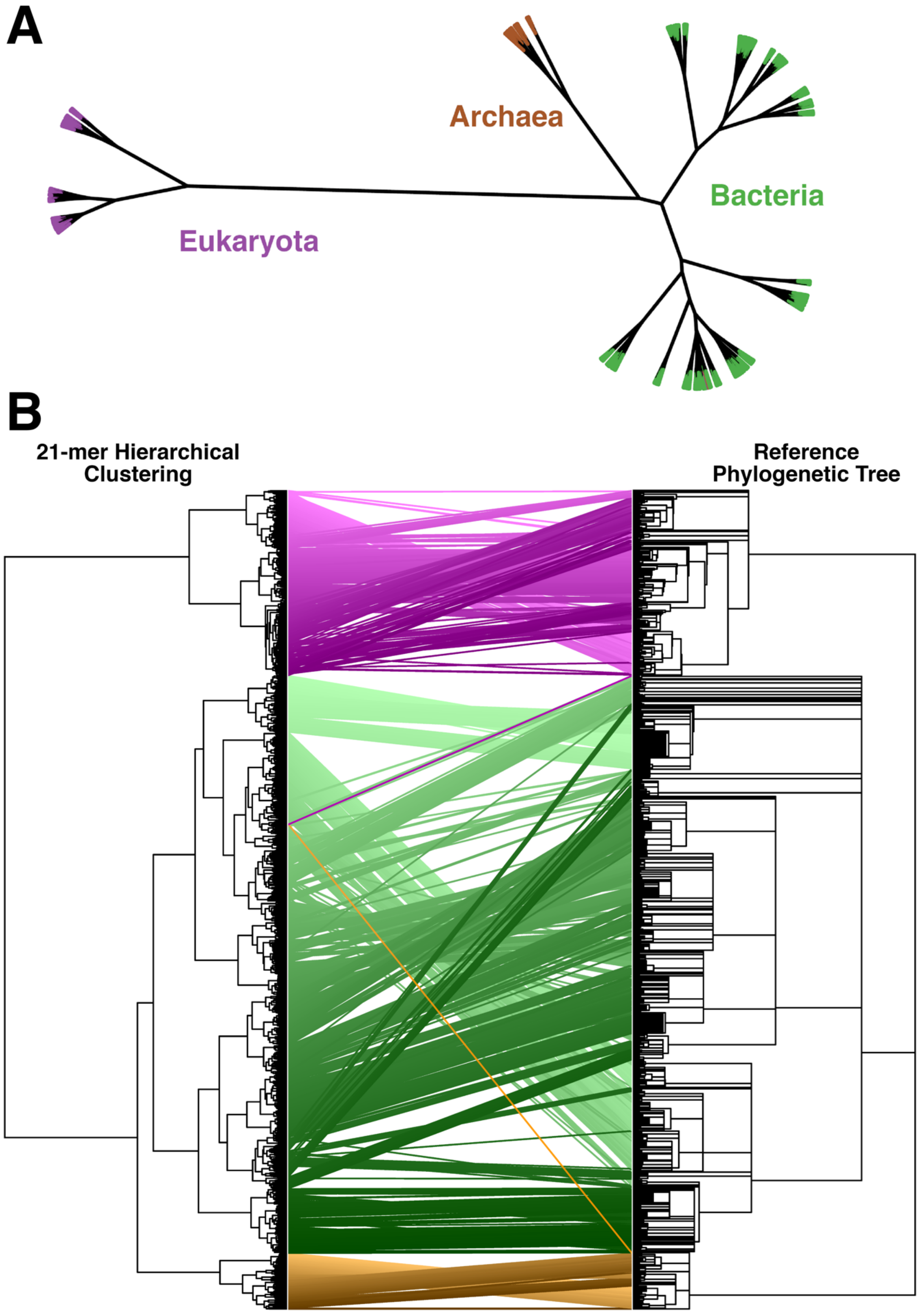

S2 Fig. Large-scale visualization of hierarchical clustering versus reference phylogenetic tree shows many comparable large subtrees.
