# Supplementary material for: Large-scale k-mer-based analysis of the informational properties of genomes, comparative genomics and taxonomy
Source: PLoS One. 2021 Oct 14;16(10):e0258693. doi: 10.1371/journal.pone.0258693 (PMC8516232; doi:10.1371/journal.pone.0258693)
Supplement: S3 Fig — The Mammalian class subtree (left) from the hierarchical clustering of 1634 genera representatives by 21-mer Jaccard similarity (Fig 4, group 9) and the corresponding reference phylogenetic tree (right) were plotted in a tanglegram. Polytomies in the reference phylogenetic tree were fully resolved using intermediate taxon labels from the database, and in few cases from the literature (see Methods for further details). Identical subtrees and their linking lines are colored (different colors). Non-identical branches/leaves and their linking lines are shown in black. Most subtrees, representing order and family phylogeny, were identically represented in the hierarchical clustering and some branches only had very minor differences, for example the connection between Pongo and Nomascus genera within the Hominoidea superfamily. (PDF) [file pone.0258693.s003.pdf]

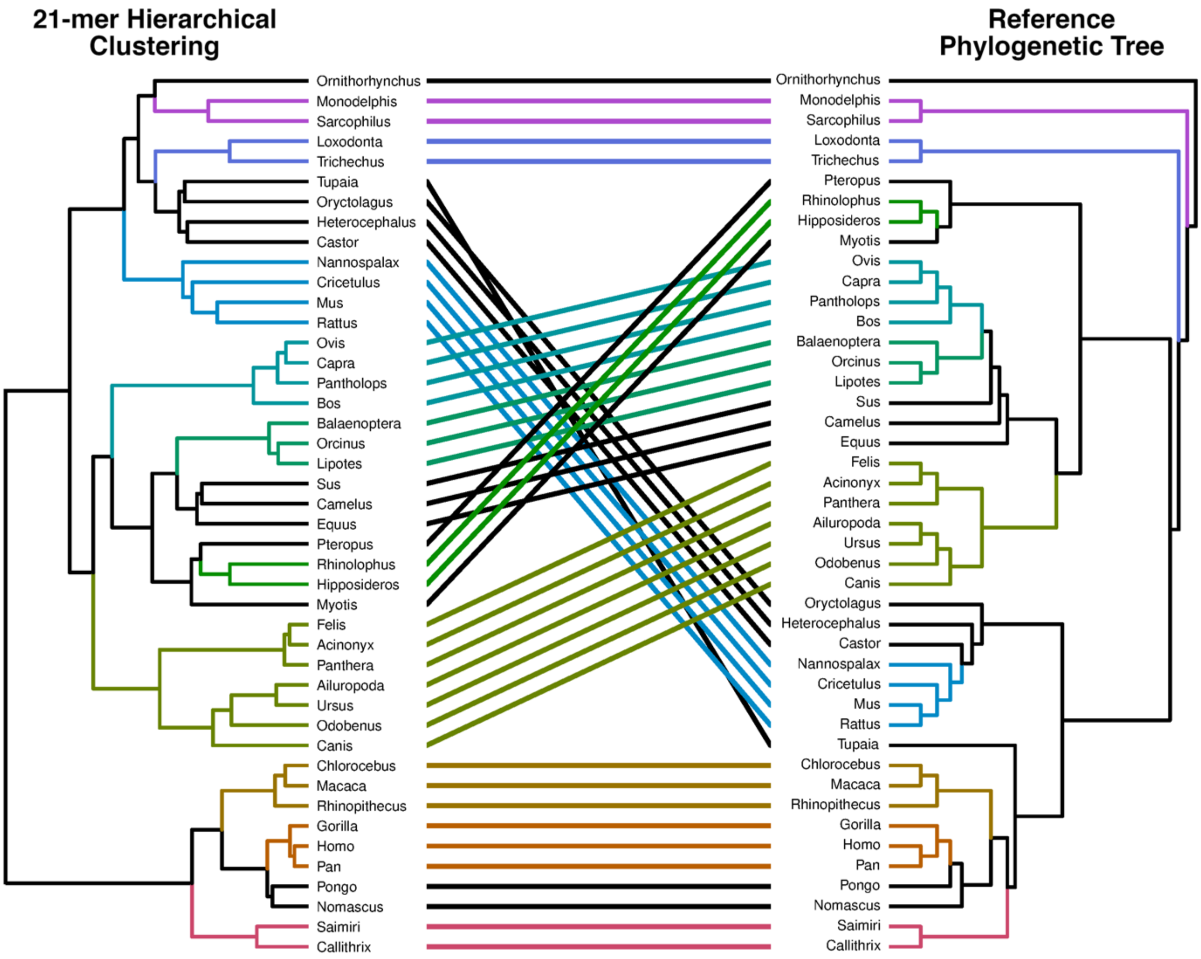

S3 Fig. Mammalian order and family subtree topology derived from hierarchical clustering is very similar compared to the reference phylogenetic tree.
